# Isolated right ventricular takotsubo cardiomyopathy presenting as acute right ventricular failure: A case report

**DOI:** 10.1016/j.heliyon.2023.e17607

**Published:** 2023-06-23

**Authors:** Hiroyuki Yamamoto, Haruna Akiba, Hiroshi Inagawa, Yasusei Okada

**Affiliations:** a Department of Cardiovascular Medicine, Showa General Hospital, Tokyo, Japan; b Department of Cardiovascular Medicine, Narita-Tomisato Tokushukai Hospital, Chiba, Japan; c Department of Critical Care and Emergency Medicine, Showa General Hospital, Tokyo, Japan

**Keywords:** Takotsubo cardiomyopathy, Isolated right ventricular takotsubo cardiomyopathy, Acute right ventricular failure, Cardiogenic shock, Echocardiography

## Abstract

**Background:**

Takotsubo cardiomyopathy (TTC) is a non-ischaemic cardiomyopathy, characterized by transient wall motion abnormalities of the left ventricle. Although biventricular involvements are common with a poor prognosis, isolated right ventricular (RV) involvement of TTC is a rare, and its diagnosis remains challenging.

**Case presentation:**

We encountered a case of isolated RV-TTC presenting as acute RV failure, with progression to cardiogenic shock requiring intensive treatment. Conflicting echocardiographic findings of RV asynergy with RV enlargement despite normal left ventricular wall motion and mild tricuspid regurgitation led to the correct diagnosis. Finally, the patient showed complete recovery with normalization of cardiac structure and function.

**Conclusions:**

This case underscores the clinical significance of considering isolated RV-TTC as a new distinct variant form of TTC in terms of presentation, diagnostic findings, differential diagnosis, treatment strategy, and prognosis.

## 1 Background

Acute right ventricular failure (ARVF) is a heterogeneous clinical syndrome, characterized by elevated right ventricular (RV) filling pressure and reduced RV output [1]. ARVF can have a variable presentation, ranging from asymptomatic to systemic congestion or serious complications such as cardiogenic shock, and is a significant prognostic determinant of various diseases. The underlying pathophysiology of ARVF is so diverse that a specific treatment strategy dependent on the respective aetiology is needed for proper management.

## 2 Case presentation

A 77-year-old man was transferred to the emergency department due to coma with a one-week history of anorexia and leg edema. On admission, vital signs included: Japan Coma Scale, III–300; blood pressure, 95/65 mmHg; temperature, 36.0 °C; heart rate, 80 beats/min. Physical examination revealed jugular venous distension, systemic edema, cold and clammy extremities, and bilateral diminished vesicular breath sounds. Severe cervical-thoracic kyphosis was noted. A chest radiograph revealed diffuse ground-glass opacities in both lungs. Arterial blood gas analysis (using Bag-valve-mask with 10 L/min O2 flow) revealed a pH of 7.18, pCO2 greater than 70 mmHg, and lactate levels of 4.0 mmol/L, indicating severe hypercapnic respiratory failure, requiring intubation and mechanical ventilation. Moreover, he received infusions of noradrenaline (0.04 μg/kg/min) and milrinone (0.125 μg/kg/min) because of progressive hemodynamic instability, that required intensive care for close monitoring.

**Fig. 1:**
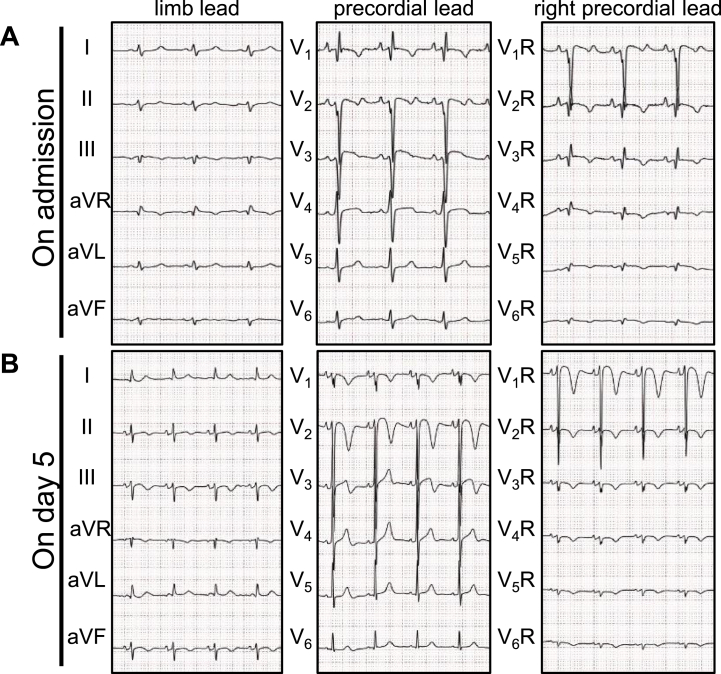
Serial 12-lead electrocardiograms with right precordial leads (A, on admission; B, on day 5).

**Fig. 2:**
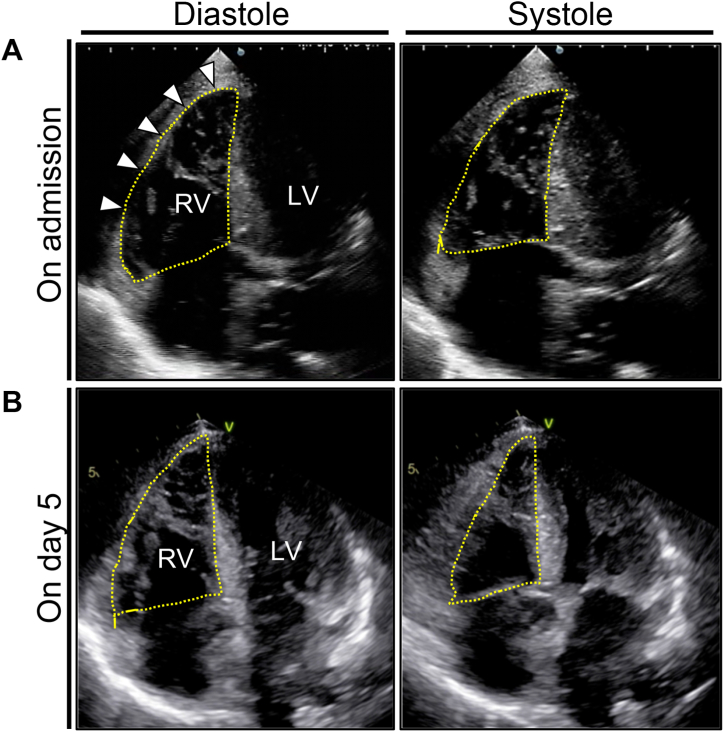
Serial echocardiography (A, on admission; B, on day 5) Apical four-chamber views of the heart during diastole and systole. Note the severely reduced systolic function of the apico-mid right ventricular free wall with right ventricular enlargement (arrowheads). LV, left ventricle; RV, right ventricle.

Laboratory findings revealed neutrophilic leukocytosis, elevated C-reactive protein levels, severe hepatorenal dysfunction, as well as disseminated intravascular coagulation. We tentatively diagnosed acute respiratory failure owing to aspiration pneumonia and severe kyphosis, and initial empirical therapy with broad-spectrum antimicrobials was initiated. In addition, mildly elevated levels of serum cardiac troponin T (0.365 ng/mL, reference: <0.014 ng/mL) and brain natriuretic peptide (183 pg/mL, reference: <18.4 pg/mL) were found. Electrocardiogram (ECG) revealed sinus tachycardia, changes of incomplete right bundle-branch block (RBBB) in V1, T-wave inversion in V1–2 and right precordial leads (V1R–V5R), and R/S > l in V1, V2R, and V3R (Fig. 1A). Echocardiography revealed severely reduced systolic function of the apico-mid RV free wall with RV enlargement; RV basal diameter = 58 mm (reference: ≤40 mm), tricuspid annular plane systolic excursion (TAPSE) = 12.3 mm (reference: ≥16 mm), and fractional area change (FAC) = 8.5% (reference: ≥35%), whereas left ventricular (LV) systolic function was fully preserved (Fig. 2A; Videos S1 and S2 in the Data Supplement). Note the mild tricuspid regurgitation (TR), which made pulmonary hypertension unlikely. Therefore, a corrected diagnosis of ARVF was made. Contrast-enhanced chest computed tomography confirmed signs of pneumonia in both lungs, but excluded pulmonary vascular or left-sided cardiac diseases (Fig. S1 in the Data Supplement).

On day 5, new giant T-wave inversion and extreme QT prolongation in the right precordial leads were documented (Fig. 1B). Follow-up echocardiography revealed full resolution of RV function as well as of wall motion abnormalities (WMAs); RV basal diameter = 44 mm, TAPSE = 19 mm, and FAC = 40% (Fig. 2B; Videos S3 and S4 in the Data Supplement). The patient's clinical condition progressively improved with resolution of hemodynamic instability, resulting in withdrawal from intensive care therapy.

**Fig. 3:**
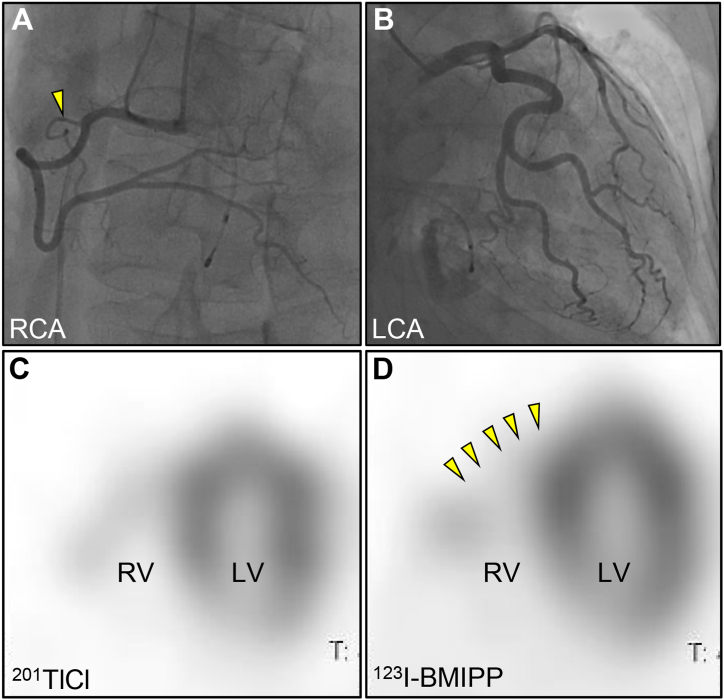
Coronary angiography and dual-isotope myocardial SPECT Coronary angiography was unremarkable including a right ventricular branch (arrowhead) (A, B). Myocardial ^201^TlCl and ^123^I-BMIPP SPECT images (long axis vertical view) (C, D). Myocardial perfusion of 201TlCl is preserved, whereas 123I-BMIPP is reduced in the apico-mid region of the RV (arrowheads), corresponding to the wall motion abnormalities identified on initial echocardiography. BMIPP, (β-methyl-*p*-Iodophenyl-Pentadecanoic Acid); LCA, left coronary angiography; LV, left ventricle; RCA, right coronary angiography; RV, right ventricle; SPECT, single-photon emission computed tomography.

Next, a detailed diagnostic work-up for ARVF was performed. Coronary angiography and vasospasm provocation test with acetylcholine were unremarkable (Fig. 3A and B). Dual-isotope myocardial single-photon emission computed tomography (SPECT) revealed that, myocardial fatty acid metabolism was more severely impaired than myocardial perfusion (arrowheads) even after resolution of RVWMAs (Fig. 3C and D). This discrepancy strongly suggested stunned myocardium. Based on these findings, the final diagnosis of an isolated right ventricular takotsubo cardiomyopathy (RV-TTC) was made.

Although he had persistent hypercapnic respiratory failure caused by severe kyphosis requiring a transient tracheostomy, the process of intensive rehabilitation enabled him eventually to wean off the ventilator without neurological complications. On day 46, he was transferred to a nursing home for further rehabilitation.

## 3 Discussion

Herein, we describe a rare case of isolated RV-TTC leading to ARVF triggered by pneumonia.

Takotsubo cardiomyopathy (TTC) is characterized by transient LV apical ballooning without significant epicardial coronary artery disease [2,3]. TTC, commonly triggered by emotional or physical stress, predominantly affects postmenopausal women and often shares common characteristics with acute coronary disease and acute left-sided heart failure. In addition to the classical form of TTC, several variant forms of TTC are known, namely mid-ventricular type (14.6%), basal type (2.2%), and focal type (1.5%) [4]. Moreover, biventricular cases, which account for one-third TTC cases, have significantly higher in-hospital complication rates and poorer prognosis [[5], [6], [7]]. However, isolated RV-TTC is an extremely rare disease and our case may provide the following five valuable clinical lessons.

Firstly, most cases of isolated RV-TTC are elderly females and often triggered by physical stress [[8], [9], [10], [11]]. The symptoms are non-specific e.g. dyspnea, and therefore are unlikely to raise suspicion of the disease. Similarly, in our case, pneumonia might have triggered the onset of isolated RV-TTC, and additional factors such as male sex and coma might have made early diagnosis difficult.

Secondly, the initial ECG revealed changes of incomplete RBBB in lead V1, and slight ST-segment elevation in V2–4. RV involvement is confirmed by the presence of slight ST-segment elevation with T-wave inversion in the right precordial leads (V2R–V5R).

It has been proposed that obvious ST-segment elevation in anterior precordial leads other than V1 is characteristic of the classical forms of TTC [12], because the lack of ST-segment elevation in lead V1 reflects the fact that the WMAs in the TTC does not extend to the region looked at by lead V1 (the LV basal septum). In fact, the lack of ST-segment elevation in lead V1 was useful for differentiating TTC from acute anterior myocardial infarction, of which the clinical manifestations are similar to those of TTC. (94% sensitivity, 71% specificity, and 73% predictive accuracies). Conversely, most cases of isolated RV-TTC have ECG findings that involve lead V1: RBBB, R/S > 1, RSR’, or QR in V1; T-wave inversion in V1–3, V2–4, or V3R–5R; discrete ST-segment elevation in V1–2. Thus, ECG findings in precordial lead V1 or right precordial leads might be valuable tips for spotting isolated RV-TTC.

Thirdly, echocardiography was useful in reaching the correct diagnosis. Compared to the left ventricle, the thin-walled right ventricle is less forceful and plays a central role in the high-compliance and low-resistance pulmonary circulation. Given the inability to adapt to rapid changes in RV afterload, predominant causes of ARVF include diseases leading to increased RV afterload (e.g., acute pulmonary embolism, hypoxia, and left-sided disease). Typical echocardiographic findings include RV dilation, reduced RV contraction, and inevitably severe TR reflecting pulmonary hypertension. However, in our case, RV dysfunction and RV dilation were notable despite the absence of significant TR, findings suggesting that the RV myocardium itself was the primary site of the lesion. These conflicting findings might be a crucial red flag for the diagnosis of this rare entity.

Fourthly, the dual-isotope SPECT imaging was also useful in reaching the definitive diagnosis. In our case, the findings of SPECT suggested that myocardial fatty acid metabolism was more severely impaired than myocardial perfusion in the region corresponding to the RVWMAs. Although the exact mechanism of TTC remains elusive, similarly to classical forms of TTC, this discrepancy suggestive of myocardial stunning was helpful in the confirmation of isolated RV-TTC [13].

Finally, our patient developed RV cardiac shock, requiring intensive treatment including vasopressors and an inotrope. Conservative treatment and relief of emotional or physical stress are the mainstream of management of typical TTC, yet isolated RV-TTC often exhibits severe clinical features such as cardiac shock and severe heart failure, requiring intensive treatment [10,11], so care is needed.

Therefore, isolated RV-TTC differs significantly from typical TTC in terms of clinical presentation, diagnostic findings, differential diagnosis, treatment strategy, and prognosis. All clinicians should recognize this rare clinical entity as a differential diagnosis of ARVF. The presence of LVWMAs is not a pathognomonic sign for the diagnosis. Comprehensive cardiac evaluation including analysis of RV function using echocardiography or cardiac magnetic resonance imaging is recommended [14].

## 4 Conclusions

Isolated RV-TTC is a rare variant of TTC, but may induce ARVF, progressing to cardiogenic shock. Therefore, clinicians should be aware of the clinical significance, and comprehensive cardiac evaluation of right ventricle is critical for early diagnosis and treatment.

## Author contribution statement

All authors listed have significantly contributed to the investigation, development and writing of this article.

## Data availability statement

No data was used for the research described in the article.

## Funding statement

This research did not receive any specific grant from funding agencies in the public, commercial, or not-for-profit sectors.

## Declaration of competing interest

The authors declare that they have no known competing financial interests or personal relationships that could have appeared to influence the work reported in this paper.

## References

[1] Harjola Veli-Pekka, Mebazaa Alexandre, Čelutkienė Jelena, Bettex Dominique, Bueno Hector, Chioncel Ovidiu (2016). Contemporary management of acute right ventricular failure: a statement from the heart failure association and the working group on pulmonary circulation and right ventricular function of the European society of cardiology. Eur. J. Heart Fail..

[2] Tsuchihashi K., Ueshima K., Uchida T., Oh-mura N., Kimura K., Owa M. (2001). Angina Pectoris-Myocardial Infarction Investigations in Japan. Transient left ventricular apical ballooning without coronary artery stenosis: a novel heart syndrome mimicking acute myocardial infarction. Angina Pectoris-Myocardial Infarction Investigations in Japan. J. Am. Coll. Cardiol..

[3] Bybee K.A., Kara T., Prasad A., Lerman A., Barsness G.W., Wright R.S. (2004). Systematic review: transient left ventricular apical ballooning: a syndrome that mimics ST-segment elevation myocardial infarction. Ann. Intern. Med..

[4] Templin C., Ghadri J.R., Diekmann J., Napp L.C., Bataiosu D.R., Jaguszewski M. (2015). Clinical features and outcomes of takotsubo (stress) cardiomyopathy. N. Engl. J. Med..

[5] Singh Kuljit, Neil Christopher J., Nguyen Thanh Ha, Stansborough Jeanette, Chong Cher-Rin, Dawson Dana (2014). Dissociation of early shock in takotsubo cardiomyopathy from either right or left ventricular systolic dysfunction. Clin. Trial Heart Lung Circ..

[6] Haghi D., Athanasiadis A., Papavassiliu T., Suselbeck T., Fluechter S., Mahrholdt H. (2006). Right ventricular involvement in Takotsubo cardiomyopathy. Eur. Heart J..

[7] Kagiyama N., Okura H., Tamada T., Imai K., Yamada R., Kume T. (2016). Impact of right ventricular involvement on the prognosis of takotsubo cardiomyopathy. Eur. Heart J. Cardiovasc. Imaging.

[8] Mrdovic I., Kostic J., Perunicic J., Asanin M., Vasiljevic Z., Ostojic M. (2010). Right ventricular takotsubo cardiomyopathy. J. Am. Coll. Cardiol..

[9] Stähli B.E., Ruschitzka F., Enseleit F. (2011). Isolated right ventricular ballooning syndrome: a new variant of transient cardiomyopathy. Eur. Heart J..

[10] Chandorkar A.1, Codolosa J.N., Lippmann M.L., Pressman G.S., Sta Cruz J.P. (2014). Recurrent right ventricular takotsubo cardiomyopathy in a patient with recurrent aspiration. Echocardiography.

[11] Elikowski W., Małek-Elikowska M., Różańska P., Fertała N., Zawodna M. (2016). Isolated right ventricular takotsubo cardiomyopathy: a case report and literature review. Pol. Merkur. Lek..

[12] Kosuge Masami, Ebina Toshiaki, Hibi Kiyoshi, Morita Satoshi, Okuda Jun, Iwahashi Noriaki (2010). Simple and accurate electrocardiographic criteria to differentiate takotsubo cardiomyopathy from anterior acute myocardial infarction. J. Am. Coll. Cardiol..

[13] Kurisu Satoshi, Inoue Ichiro, Kawagoe Takuji, Ishihara Masaharu, Shimatani Yuji, Nishioka Kenji (2003 5). Myocardial perfusion and fatty acid metabolism in patients with tako-tsubo-like left ventricular dysfunction. J. Am. Coll. Cardiol..

[14] Vernay C., Karsenty C., Redheuil A., Soulat G., Iserin L. (2018). Systemic right ventricular takotsubo cardiomyopathy. Eur. Heart J..

