# Supplementary figures and images for: Isolated right ventricular takotsubo cardiomyopathy presenting as acute right ventricular failure: A case report
Source: Heliyon. 2023 Jun 23;9(6):e17607. doi: 10.1016/j.heliyon.2023.e17607 (PMC10320258; doi:10.1016/j.heliyon.2023.e17607)

## Slide 1
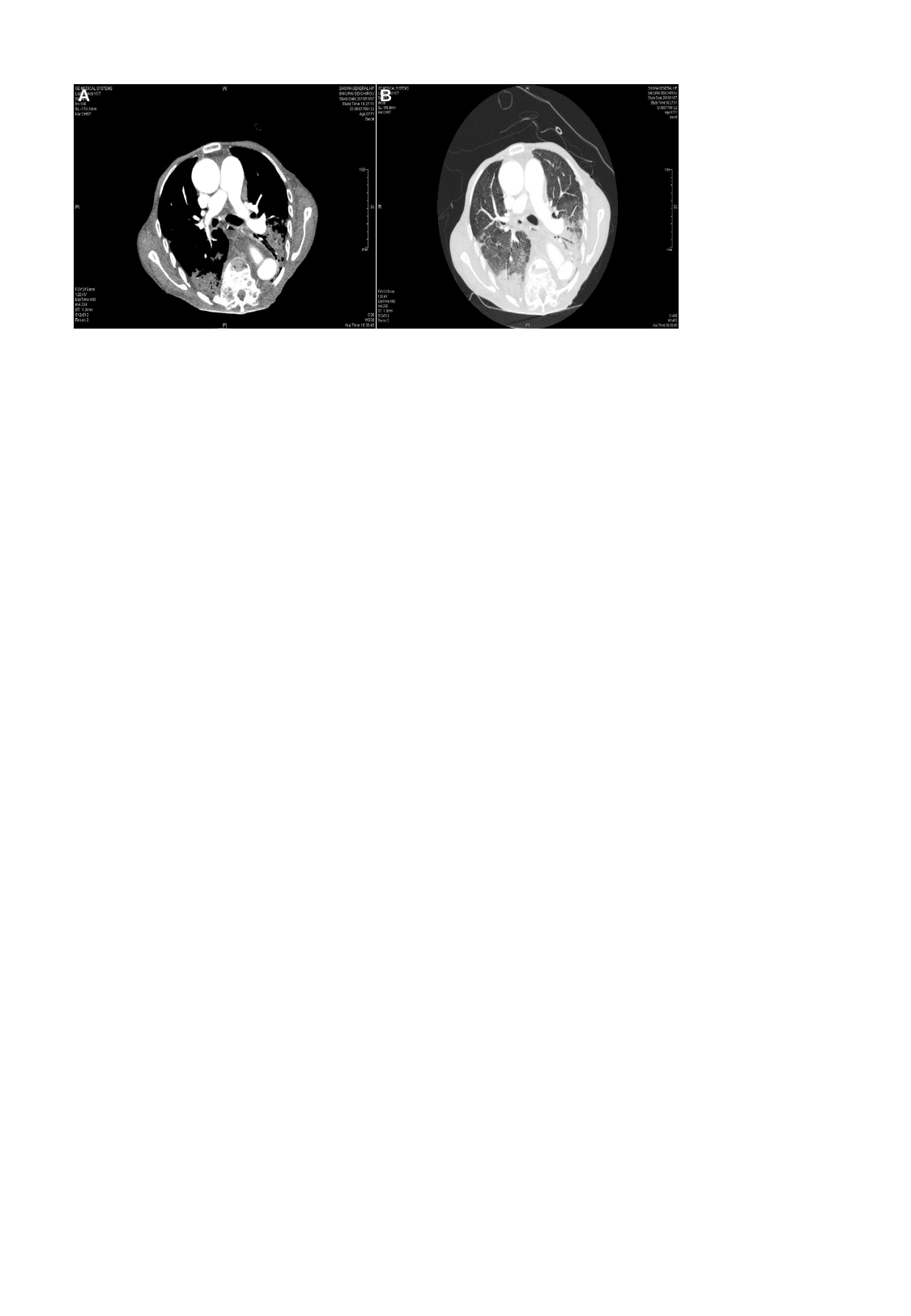

A
B

Supplement: Multimedia component 5 [file mmc5.pptx]
